# Supplementary material for: Patterns of Intron Gain and Loss in Fungi
Source: PLoS Biol. 2004 Nov 30;2(12):e422. doi: 10.1371/journal.pbio.0020422 (PMC532390; doi:10.1371/journal.pbio.0020422)
Supplement: Table S1 — Also available at http://genes.mit.edu/NielsenEtAl/. (4.3 MB ZIP). [file pbio.0020422.st001.zip › NielsenEtAl/html/477.html]

AN5146.1.NCU07362.1.MG00335.1.FG01027.1


```
 CLUSTAL W (1.82) Multiple Sequence Alignments - Introns Inserted


Sequence 1: NCU07362.1	520 aa
Sequence 2: MG00335.1	510 aa
Sequence 3: FG01027.1	488 aa
Sequence 4: AN5146.1	475 aa
Alignment Length: 521 aa
Number Identitical Residues: 249 aa
Alignment Score (without introns) 12332


MG00335.1 	---MAKVFDAEEV1AKHNTPESCWVILYGDVYD~VTDFVPSHPGGSRIILQLAGRDATEE
NCU07362.1	MTKPPKVFTAEEV1AKHRSPKSCWVVLYGNVYD~VTEFLPSHPAGDKIILQLAGRDATTE
FG01027.1 	---MAKVFDAAEV1AKHNTPESCWVILYGKVYD~VTEFLPSHPGGKKIILKLAGKDATEE
AN5146.1  	---MGKVFDAAEV1AKHNTKDSCWVILYG---K0VTDFLSEHPGGSKIILKLSGQDATEE
          	     *** * ** ***.: .****:***   . **:*:..**.*.:***:*:*:*** *

MG00335.1 	YDPVHPPGTLEENLKPEAKLGTVDPASLERLRKAN---QAQAAANVTGRTNEGSSGNAEE
NCU07362.1	YDPIHPPGTLEENLKPEAKLGIIDDASLAKLHQKESEARAKATGSAMGTTTHTTSAQQSE
FG01027.1 	YDPVHPPGTLEENLKPEHVLGEINPETLS---------QSQSTG------EKGVTQSSED
AN5146.1  	YDPIHPPGILEENLKPEAFLGTVDSATLP---------KPKADA------TVAQEKKEEE
          	***:**** ********  ** ::  :*          :.:: .            . .:

MG00335.1 	KKQAPPPLETLFNLDEIEEVAKQQVSRKCWAYYWSAGDDQISKVLNGRVYRDILLRPRVF
NCU07362.1	K-AAPPPLHTLLNFDEIEAAAKTQVSKKCWSYYFSAADDLYTKTHNTRAFRDILLRPRVF
FG01027.1 	Q---PPPLASLMNLDEIEQEATKRISKKAWAYYFSAADDLFSKSYNNHVFKNILIRPRVF
AN5146.1  	D----VPMEALLNMDDIEQLATKKVSKKAWAYYYSASDDKITKQFNTDVYRAITLRPRVF
          	.     *: :*:*:*:**  *. ::*:*.*:**:**.**  :*  *  .:: * :*****

MG00335.1 	VDCTSCDLSTTMLGNKVGTPLYVSPAAMARLAHPDGEHGIAKGISSFGGLQIVSNNASQT
NCU07362.1	IDCTKADTSTTLLGHKVGTPLYVSPAALARLAHPDGEAGIAKGISSFGAMQLVSNNASMT
FG01027.1 	VDCTACDTSTTLIGNKVGLPIFVSPAAMARLAHPDGEQGIAKACARFGAMQIVSNNASMT
AN5146.1  	IDCSKCDLDISCLGYKLGIPIYVSPAAMARLGNPAGEAGIAEACRSFGAMQIISNNASMT
          	:**: .* . : :* *:* *::*****:***.:* ** ***:.   **.:*::***** *

MG00335.1 	PEQIVEGAAPGQVFGWQLYVQNDRNKNYAMLKRIHALRDHYKFIVLTLDAPVPGKRELDE
NCU07362.1	PEQIVAEAIPGAVFGWQLYVQTTRSKSEAMLARINKLRDHFKCIVLTLDAPHPGKREHDE
FG01027.1 	PEQIIEGAKPGQIFGWQLYVQNQRDKSEAMLKRINSMRDYYKFICLTLDAPVPGKRELDE
AN5146.1  	PEQIVENAAPDQVFGWQLYVQTNRKKSEAQLARVNKLK-AIKFVVLTLDAPVPGKREDDE
          	****:  * *. :********. *.*. * * *:: ::   * : ****** ***** **

MG00335.1 	KQQFLESGMTMSAASAG--GAPKHPAGGGVGQQLFWGTAADLTWTTTLPWLAEHTDLPIV
NCU07362.1	KSNLEAAGEFVESASNAKTDAEKKPGGGGVGQQLFWGTAADLTWETTLPWLSKHTDLPIV
FG01027.1 	KQNFDYS----EPSPAS---GESKPGAGGVGQQLFFGTAADLTWKTTLPWLAAHTDLPIV
AN5146.1  	RGN--------AATGAG-------QGESGVGKQLFQGTDPTLTWRDTLPWLKKHTDLPII
          	: :         .:  .        . .***:*** ** . ***  *****  ******:

MG00335.1 	LKGIQTHEDAYLAAQYAAKY-GTVKAIILSNHGGRALDTAPPAVHTLLEIRKYCPEVFDQ
NCU07362.1	LKGIQTHEDAYLAAQYARKHPGTVKAIILSNHGGRALDTAPPAVHTLLEIRKYCPEVFGA
FG01027.1 	LKGLQAHEDAFLAAKYAPQV----KAIILSNHGGRAADTAPPAMHTLLEIRKYCPEIMSK
AN5146.1  	LKGLQTHEDAYIASLHGPQV----KGIILSNHGGRALDTAPPAVHTLLEIRKYCPEVFDK
          	***:*:****::*: :. :     *.********** ******:************::. 

MG00335.1 	IEVWVDGGIKRGTDVIKALCLGAKAVGVGRAALYGLGAGGWKGVERTFEI1LNGEMATCM
NCU07362.1	VEVWIDGGVKRGTDVVKALCLGAKAVGVGRAALWGLGAGGWQGVERTFDI1LQQEIITCM
FG01027.1 	VQIWIDGGIKRGTDVVKALCLGASGVGIGRAALFGLGAGGQAGVERTLEI1LEAETATCM
AN5146.1  	LEVLVDGGIRRGTDVVKALCLGAKAVGIGRPALWGLGAGGVAGVKRTLQI1LADETSTAM
          	::: :***::*****:*******..**:**.**:******  **:**::* *  *  *.*

MG00335.1 	KLLGAKTVADLGPHF0VNYRKVERDIYDGPSGLDSK--SWAKAKI
NCU07362.1	KLLGAKTVNDLGPRF0INTRKVERDIFDGDAGLDKNGLWTSRAKL
FG01027.1 	RLLGAKNISELGPRF0VNARQVERDIYDGDAGLDRQG-LWTKSKL
AN5146.1  	RLLGCERVEQLGPHH0VNTRVVEQQIYDGPPGLDYIR-SAYRAKL
          	:***.: : :***:. :* * **::*:** .***       ::*:
```
